# Clinicopathological characteristics and prognosis of adolescents and young adults with gastric cancer after gastrectomy: a propensity score matching analysis

**DOI:** 10.3389/fonc.2023.1204400

**Published:** 2023-08-18

**Authors:** Hongwu Chu, Xiaoyan Chen, Xin Liu, Cuncan Deng, Bo Bi, Yulong He, Mingyu Huo, Changhua Zhang

**Affiliations:** ^1^ Guangdong Provincial Key Laboratory of Digestive Cancer Research, The Seventh Affiliated Hospital of Sun Yat-sen University, Shenzhen, China; ^2^ Department of Gastrointestinal Surgery, The First Affiliated Hospital of Sun Yat-Sen University, Guangzhou, China; ^3^ Qingdao Medical College, Qingdao University, Qingdao, China

**Keywords:** gastric cancer, age, young, clinicopathological characteristics, prognosis

## Abstract

**Background:**

Gastric cancer (GC) among adolescents and young adults (AYAs, aged 15-39 years) has limited data on clinicopathological characteristics and prognosis. This study aimed to compare the clinicopathological characteristics, perioperative outcomes, and long-term outcomes of AYAs and older adults (OAs, aged > 39 years) with GC who underwent curative gastrectomy.

**Methods:**

From January 1994 to June 2019, patients with GC undergoing curative gastrectomy were enrolled and divided into AYA group and OA group. The clinicopathological characteristics, treatment variables, perioperative outcomes and long-term outcomes were compared between the two groups, both before and after propensity score matching (PSM).

**Results:**

AYAs had fewer comorbid conditions and were more likely to be females, have normal carcinoembryonic antigen (CEA) levels, poorly differentiated tumors with perineural invasion, and receive adjuvant chemotherapy. AYA patients had lower incidence of postoperative complications and shorter length of postoperative hospital stay than OA patients. No significant differences in postoperative 30-day or 90-day mortality were observed between AYAs and OAs, both before and after PSM. In the entire cohort, AYAs had similar median overall survival (OS) to OAs. However, in the PSM cohort, AYAs had significantly shorter median OS. Young age (15-39 years) was an independent risk factor for OS in GC patients following gastrectomy.

**Conclusion:**

The clinicopathological characteristics were significantly different between AYA and OA patients with GC. AYA patients with GC had worse long-term prognosis than OA patients, and young age was an independent risk factor for OS in GC patients following gastrectomy.

## 1 Introduction

Gastric cancer (GC) is still a killer globally, ranking the fifth most common cancer and the fourth leading cause of cancer-related deaths (1). GC often occurs in middle-aged and elderly people, with the highest incidence among people aged 50 to 70 years (2). In the past decades, the morbidity and mortality of GC have been declining consistently across world regions (3). The morbidity of GC in adolescents and young adults (AYAs) has also shown a decreasing trend in most countries, accompanied by a decrease in mortality across almost all nations (4). However, the mortality-to-incidence ratio (MIR) of GC in AYAs ranked seventh among all cancer types, indicating a higher burden and poorer prognosis compared to other cancer types in in this age group (4). In addition, in the past two decades, GC in young patients has shown an increasing trend in invasion depth, lymph node metastasis and the proportion of poorly differentiated adenocarcinoma, which indicates that GC in young patients is developing into a more serious disease state (5). A large number of studies demonstrated that compared with the elderly patients, young patients with GC have unique clinicopathological characteristics, such as a higher proportion of female patients and poorer differentiation (5–9). Currently, the prognosis of young GC patients is still controversial. Some studies reported that the prognosis of young patients with GC is significantly worse than that of elderly patients (10, 11). On the contrary, some studies have shown that the long-term survival of young patients with GC is comparable to that of elderly patients (2, 8, 12). The National Cancer Institute of the United States defines cancer in AYAs as diagnoses that occurs among those aged 15 to 39 years (13). AYAs have been identified as a population different from children and middle-aged and elderly people (14). Cancers in AYAs are different from those in other age groups in internal and external risk factors, tumor biology, and prognosis (15). Compared with elderly patients, AYAs has a higher risk of GC-specific death (16). However, the clinicopathological features, perioperative outcomes and long-term outcomes of GC in AYA patients are poorly described. The aim of this retrospective cohort study was to compare the clinicopathological characteristics of AYA patients and older adults (OA) patients with GC, and to determine prognostic factors for AYA patients with GC.

## 2 Materials and methods

### 2.1 Patient selection

From January 1994 to June 2019, patients with GC who underwent gastrectomy in the First Affiliated Hospital of Sun Yat-sen University were identified. The data of the patients were retrospectively analyzed, and the patients were divided into two groups based on age, among them, those aged 15-39 years were AYAs group, and those aged > 39 years were OAs group. Postoperative pathological examination confirmed the diagnosis of GC. The exclusion criteria are as follows: age less than 15 years old, previous history of malignant tumor, underwent preoperative neoadjuvant chemotherapy, recurrent GC, underwent R1 or R2 resection, distant metastasis, loss of follow-up, and incomplete case data. This study was conducted in accordance with the Helsinki Declaration and was approved by the Institutional Review Board of the Seventh Affiliated Hospital of Sun Yat-sen University. All the patients included in the study provided written informed consent.

### 2.2 Clinicopathological features, treatment variables, and perioperative outcomes

The clinicopathological features of patients included age, sex, clinical manifestations, family history of cancer, comorbid conditions, preoperative carcinoembryonic antigen (CEA) level, ascites, maximum tumor size, tumor location, Borrmann type, tumor differentiation, TNM stage, vascular invasion, lymphatic invasion and perineural invasion. CEA level >5μg/L was considered to be positive. The eighth edition of the American Joint Committee on Cancer TNM staging system was used to identified the TNM stage (17). Treatment variables included operation time, intraoperative blood transfusion, gastrectomy type, lymphadenectomy type, resection margin, and adjuvant chemotherapy. Perioperative outcomes included postoperative complications, length of postoperative hospital stay, postoperative 30-day and 90-day mortality. Postoperative complications was classified according to the Clavien-Dindo classification (18).

### 2.3 Postoperative follow−up

The patients were followed up every 2 months during the first 2 years, every 6 months for the subsequent 3 years, and then annually thereafter. The postoperative surveillance strategies included physical examination, serum tumor markers, chest X-ray, gastroscopy, and abdominal contrast-enhanced computed tomography (CT) scan.

### 2.4 Study endpoint and propensity score matching (PSM) analysis

The endpoint of the study was the overall survival (OS) of the patient, and the OS was calculated from the date of undergoing surgery until death from any cause or the last follow-up. The AYA patients and OA patients were matched by propensity score matching (PSM) described by Rubin and Rosenbaum (19, 20), which was performed using R software version 4.2.2. The individual propensity score was calculated given the covariates of sex, family history of cancer, comorbid conditions, CEA, operation time, gastrectomy type, intraoperative blood transfusion, ascites, tumor size, tumor location, Borrmann type, tumor differentiation, TNM stage, T status, N status, vascular invasion, lymphatic invasion, perineural invasion, postoperative complications, and adjuvant chemotherapy using a logistic regression model. In order to minimize the conditional bias, we performed 1:1 nearest neighbor matching without replacement. Nearest neighbor matching is based on greedy matching algorithm, which matches each patient in the treatment group with the control patient who has the closest propensity score. For each AYA patient, match one OA patient with the lowest tendency score distance. We tested a variety of caliper widths and used standardized mean differences to check the balance of covariate distribution between two groups. Finally, we found that 0.1 caliper meets the requirements of preferable homogeneity and small sample loss.

### 2.5 Statistical analysis

Statistical analysis was performed using IBM SPSS Statistics 26.0 (SPSS, Inc., Chicago, IL, USA). Continuous variables were expressed as mean ± standard deviation (SD) or median (interquartile range, IQR), and categorical variables were expressed as numbers (percentages). Continuous variables were compared by using Student t-test or Mann-Whitney U test, as appropriate. Pearson’s Chi-square test or Fisher’s exact test was used for the comparison of categorical variables, as appropriate. Kaplan-Meier curve generated by log-rank test was used to compare the OS of AYA patients and OA patients. Univariate and multivariate Cox proportional hazard regression analyses were performed to determine independent predictors of OS in GC patients. Variables considered to be potentially important for univariate Cox proportional regression analysis (p < 0.1) were included in multivariate Cox proportional regression analysis. All tests were two-tailed and p values < 0.05 were considered as the accepted level of statistical significance.

## 3 Results

**Figure 1:**
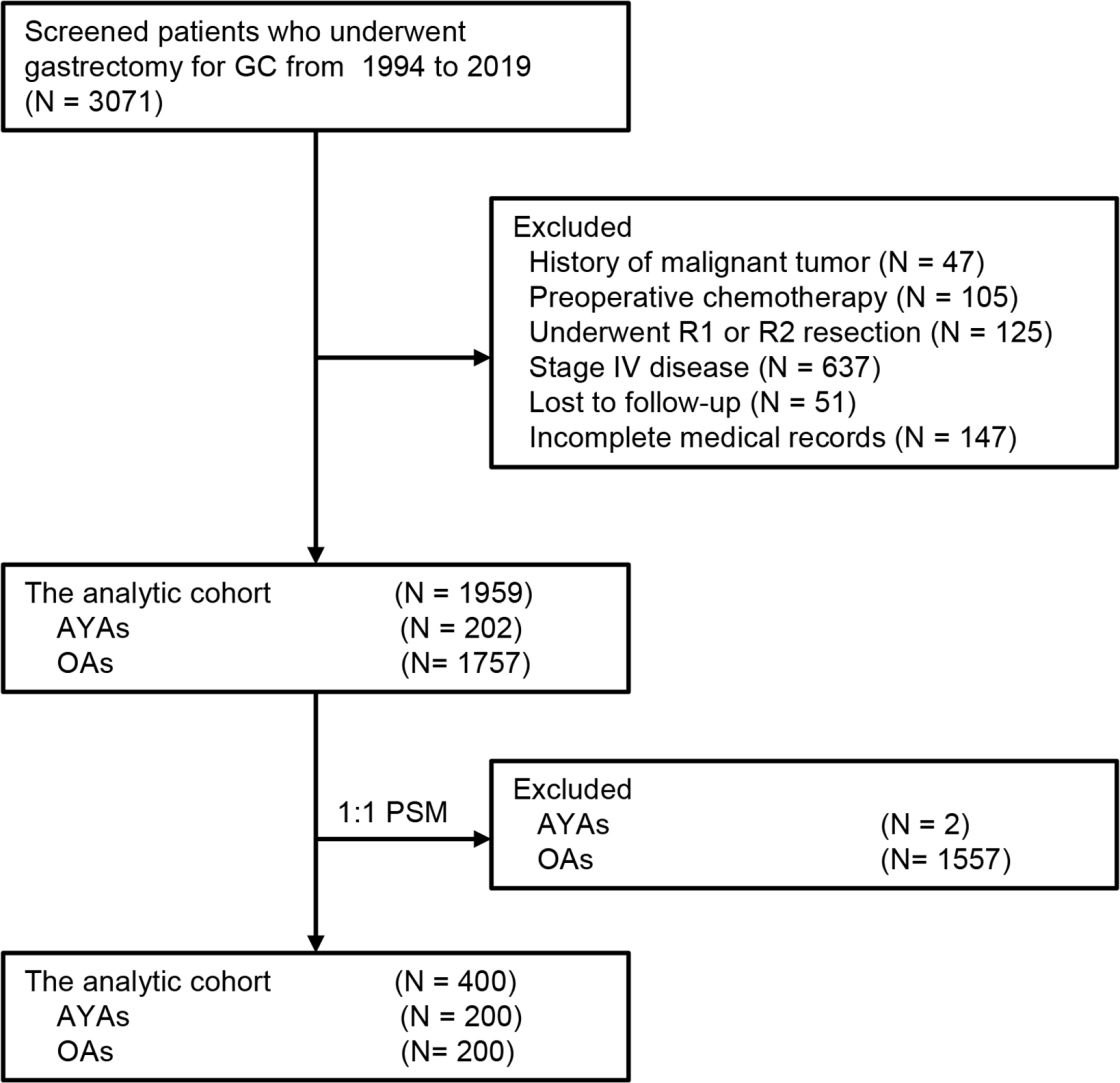
Selection of the study population.

**Figure 2:**
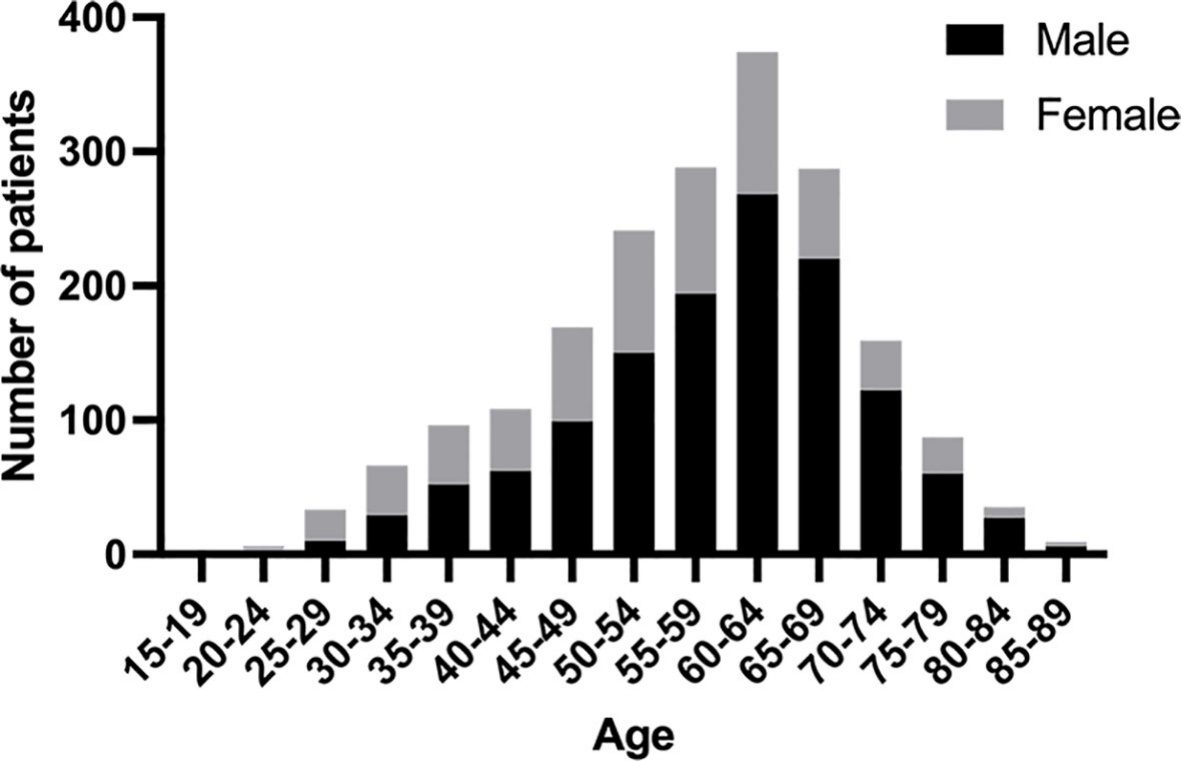
Age and sex histograms for all patients included in the study.

During the study interval, a total of 3071 patients underwent gastrectomy for GC. Of those, 1959 patients who met the pre-determined inclusion criteria were included in the analysis (**Figure 1**). Among these 1959 patients, the median age was 59 (range: 19-87) years, of which 202 (10.3%) were classified as AYAs and 1757 (89.7%) were classified as OAs. The histogram of the age and sex distribution of 1959 patients is showed in **Figure 2**. PSM was used to create 200 pairs of patients who were AYAs or OAs.

### 3.1 Patient characteristics and perioperative outcomes

The clinicopathological features, treatment variables, and perioperative outcomes of AYA and OA patients before and after PSM are presented in **Table 1**. The proportion of female in AYA patients was significantly higher than that in OA patients (53.5 vs 31.2%, p < 0.001). Compared with OA patients, there are less AYA patients presented with abdominal distension (15.8% vs. 24.6%, p = 0.006), dysphagia (2.5% vs. 8.6%, p = 0.004) and weight loss (14.9% vs. 26.9%, p < 0.001). Hypertension (0% vs. 13.0%, p < 0.001) and diabetes (0% vs. 5.7%, p = 0.001) were significantly less common in AYA patients, and no AYA patients had two or more comorbid conditions. AYA patients were more often had a normal preoperative CEA level (CEA >5μg/L, 7.9% vs. 16.8%, p = 0.001), poorly differentiation (85.1% vs. 63.3%, p < 0.001), had perineural invasion (3% vs. 0.9%, p = 0.009), and underwent adjuvant chemotherapy (59.9% vs. 44.5%, p < 0.001), and less often had tumor located in the upper third of stomach (12.9% vs. 31.6%, p < 0.001). In addition, AYA patients shared some features with the OA patients, including family history of cancer, operation time, gastrectomy type, intraoperative blood transfusion, ascites, tumor size, Borrmann type, TNM stage, T status, N status, vascular invasion, lymphatic invasion, and lymphadenectomy type.

**Table 1:** Clinicopathologic characteristics, operative variables, and perioperative outcomes.

Clinical characteristics	Before PSM (N = 1959)			After PSM (N = 400)		
	AYA patients (N = 202)	OA patients (N = 1757)	*p* value	AYA patients (N = 200)	OA patients (N = 200)	*p* value
Age, years, mean ± SD	33.5 ± 4.5	60.1 ± 9.7	< 0.001***	33.5 ± 4.5	57.9 ± 9.6	< 0.001***
Female sex	108 (53.5%)	549 (31.2%)	< 0.001***	106 (53.0%)	113 (56.5%)	0.482
Abdominal pain	96 (47.5%)	884 (50.3%)	0.453	94 (47.0%)	104 (52.0%)	0.317
Abdominal distension	32 (15.8%)	432 (24.6%)	0.006**	32 (16.0%)	44 (22.0%)	0.126
Regurgitation	27 (13.4%)	282 (16.1%)	0.322	26 (13.0%)	30 (15.0%)	0.564
Dysphagia	5 (2.5%)	151 (8.6%)	0.004**	5 (2.5%)	9 (4.5%)	0.276
Loss of appetite	44 (21.8%)	456 (26.0%)	0.198	44 (22.0%)	44 (22.0%)	1
Weight loss	30 (14.9%)	473 (26.9%)	< 0.001***	30 (15.0%)	55 (27.5%)	0.002**
Hematochezia	31 (15.3%)	274 (15.6%)	0.927	31 (15.5%)	26 (13%)	0.474
Family history of cancer	23 (11.4%)	177 (10.1%)	0.56	23 (11.5%)	20 (10.0%)	0.628
Hypertension	0	228 (13.0%)	< 0.001***	0	2 (1.0%)	0.499
Heart disease	0	30 (1.7%)	0.061	0	1 (0.5%)	1
Diabetes	0	100 (5.7%)	0.001**	0	1 (0.5%)	1
Pulmonary disease	2 (1.0%)	34 (1.9%)	0.344	2 (1%)	1 (0.5%)	1
Comorbid conditions ≥ 2	0	61 (3.5%)	0.007**	0	0	1
CEA > 5μg/L	16 (7.9%)	296 (16.8%)	0.001**	16 (8.0%)	11 (5.5%)	0.319
Operation time > 300min	60 (29.7%)	602 (34.3%)	0.194	60 (30.0%)	50 (25.0%)	0.263
Gastrectomy type			0.847			0.543
Subtotal	114 (56.4%)	1004 (57.1%)		113 (56.5%)	119 (59.5%)	
Total	88 (43.6%)	753 (42.9%)		87 (43.5%)	81 (40.5%)	
Intraoperative blood transfusion > 200ml	49 (24.3%)	423 (24.1%)	0.954	49 (24.5%)	51 (25.5%)	0.817
Ascites	9 (4.5%)	110 (6.3%)	0.309	9 (4.5%)	11 (5.5%)	0.646
Tumor size > 5cm	52 (25.7%)	491 (27.9%)	0.508	52 (26.0%)	53 (26.5%)	0.91
Tumor site			< 0.001***			0.087
Upper	26 (12.9%)	555 (31.6%)		26 (13.0%)	40 (20.0%)	
Middle	57 (28.2%)	275 (15.7%)		55 (27.5%)	37 (18.5%)	
Lower	110 (54.5%)	880 (50.1%)		110 (55.0%)	114 (57.0%)	
Whole	9 (4.5%)	47 (2.7%)		9 (4.5%)	9 (4.5%)	
Borrmann type			0.441			0.724
I	7 (3.5%)	69 (3.9%)		7 (3.5%)	8 (4.0%)	
II	60 (29.7%)	484 (27.5%)		58 (29%)	67 (33.5%)	
III	115 (56.9%)	1077 (61.3%)		115 (57.5%)	109 (54.5%)	
IV	20 (9.9%)	127 (7.2%)		20 (10.0%)	16 (8.0%)	
Differentiation			< 0.001***			0.103
Well	8 (4.0%)	53 (3.0%)		8 (4.0%)	4 (2.0%)	
Moderate	22 (10.9%)	591 (33.6%)		22 (11.0%)	35 (17.5%)	
Poor	172 (85.1%)	1113 (63.3%)		170 (85.0%)	161 (80.5%)	
TNM stage						0.422
I	45 (22.3%)	384 (21.9%)	0.904	45 (22.5%)	46 (23.0%)	
II	72 (35.6%)	605 (34.4%)		71 (35.5%)	82 (41.0%)	
III	85 (42.1%)	768 (43.7%)		84 (42.0%)	72 (36.0%)	
T status			0.844			0.269
1	33 (16.3%)	264 (15%)		33 (16.5%)	32 (16.0%)	
2	25 (12.4%)	230 (13.1%)		25 (12.5%)	39 (19.5%)	
3	75 (37.1%)	617 (35.1%)		74 (37.0%)	71 (35.5%)	
4	69 (34.2%)	646 (36.8%)		68 (34.0%)	58 (29.0%)	
N status			0.674			0.278
0	82 (40.6%)	660 (37.6%)		81 (40.5%)	76 (38%)	
1	44 (21.8%)	446 (25.4%)		44 (22.0%)	60 (30.0%)	
2	36 (17.8%)	323 (18.4%)		36 (18.0%)	34 (17.0%)	
3	40 (19.8%)	328 (18.7%)		39 (19.5%)	30 (15.0%)	
Vascular invasion	5 (2.5%)	59 (3.4%)	0.504	5 (2.5%)	6 (3.0%)	1
Lymphatic invasion	11 (5.4%)	154 (8.8%)	0.108	11 (5.5%)	7 (3.5%)	0.335
Perineural invasion	6 (3%)	16 (0.9%)	0.009**	4 (2.0%)	2 (1.0%)	0.681
Lymphadenectomy type			1			1
< D2	2 (1.0%)	22 (1.3%)		2 (1.0%)	3 (1.5%)	
≥ D2	200 (99.0%)	1735 (98.7%)		198 (99.0%)	197 (98.5%)	
Postoperative complications	10 (5.0%)	207 (11.8%)	0.003**	10 (5.0%)	13 (6.5%)	0.519
Clavien-Dindo			0.033**			0.747
No complications	192 (95.0%)	1550 (88.2%)		190 (95.0%)	187 (93.5%)	
Grade I	9 (4.5%)	172 (9.8%)		9 (4.5%)	11 (5.5%)	
Grade II	0	3 (0.2%)		0	1 (0.5%)	
Grade III	1 (0.5%)	32 (1.8%)		1 (0.5%)	1 (0.5%)	
Adjuvant chemotherapy	121 (59.9%)	781 (44.5%)	< 0.001***	119 (59.5%)	118 (59%)	0.919
Postoperative 30-day mortality	0	11 (0.6%)	0.617	0	1 (0.5%)	1
Postoperative 90-day mortality	3 (1.5%)	24 (1.4%)	1	3 (1.5%)	1 (0.5%)	0.615
Postoperative hospital stays, days, median (IQR)	10 (7.8-12)	10 (8–13)	0.017*	10 (8–12)	11 (8-14)	0.027*

AYA, adolescent and young adult; OA, older adult; CEA, carcinoembryonic antigen; PSM, propensity score match; SD, standard deviation; IQR, interquartile range; * p < 0.05; ** p < 0.01; *** p < 0.001.

Across the entire cohort, AYA patients had lower incidence of postoperative complications than OA patients (5% vs. 11.8%, p = 0.003), and the length of postoperative hospital stay of AYA patients was also shorter than that of OA patients (median [IQR] 10 [7.8-12] vs. 10 [8-13], p = 0.017). However, the postoperative 30-day and 90-day mortality were comparable between AYA and OA patients (all p > 0.05).

After PSM, except for the clinical manifestation of weight loss, other clinicopathological characteristics, treatment variables and postoperative complications were balanced between the AYA and OA groups. Similar to the analysis for the entire cohort, the length of postoperative hospital stay of AYA patients was shorter than that of OA patients (median [IQR] 10 [8-12] vs. 11 [8-14], p = 0.027), and there were still no significant differences in postoperative 30-day and 90-day mortality between the two groups (all p > 0.05).

### 3.2 Long−term outcomes

**Figure 3:**
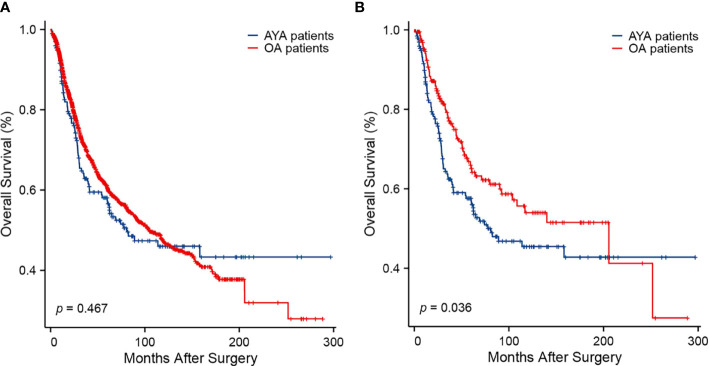
Kaplan-Meier curves of overall survival. **(A)** Before propensity score matching (*p* = 0.467). **(B)** After propensity score matching (*p* = 0.036).

The comparison of long-term outcomes between AYA and OA patients before and after PSM are presented in **Table 2**. With a median follow-up of 35.5 months, mortality was observed in 42.1% and 38.0% of AYA patients and OA patients in the entire cohort, respectively (*p* = 0.255). Among all patients in the study, the median OS of AYA patients was 80.8 months, which was similar to OA patients (104.8 months, *p* = 0.467) (**Figure 3A**). The 1-, 3-, 5-, and 10-year OS rates of AYA patients were 86.5%, 62.9%, 57.3% and 46.0% respectively, and the OS rates of OA patients were 90.9%, 71.1%, 60.8%, and 47.2%, respectively. After PSM, the mortality of AYA patients was higher than that of OA patients (42.5% vs. 32.5%, *p* = 0.039). In the PSM cohort, the median OS of AYA patients was 77.6 months, shorter than that of OA patients (205.6 months, *p* = 0.036) (**Figure 3B**). The 1-, 3-, 5-, and 10-year OS rates of AYA patients were 86.4%, 62.4%, 56.8%, and 45.5%, respectively, which were inferior to those of OA patients (94.0%, 77.8%, 65.1%, and 54.0%, respectively).

**Table 2:** Long-term outcomes before and after propensity score matching.

	Before PSM (N = 1959)			After PSM (N = 400)		
	AYA patients (N = 202)	OA patients (N = 1757)	*p* value	AYA patients (N = 200)	OA patients (N = 200)	*p* value
Death during the follow-up	85 (42.1%)	667 (38.0%)	0.255	85 (42.5%)	65 (32.5%)	0.039*
Median OS (95% CI)	80.8 (18.4 - 143.3)	104.8 (88.6 - 121.1)	0.467	77.6 (15.2 - 140.0)	205.6 (72.5 - 338.7)	0.036*
1-year OS rate, %	86.5%	90.9%		86.4%	94.0%	
3-year OS rate, %	62.9%	71.1%		62.4%	77.8%	
5-year OS rate, %	57.3%	60.8%		56.8%	65.1%	
10-year OS rate, %	46.0%	47.2%		45.5%	54.0%	

AYA, adolescent and young adult; OA, older adult; OS, overall survival; CI, confidence interval; PSM, propensity score match; * p < 0.05.

### 3.3 Prognostic analyses

Univariate and multivariate Cox proportional hazard regression analyses were performed in the cohort before and after PSM. In the cohort before PSM, family history of cancer, CEA level, intraoperative blood transfusion, ascites, tumor size, tumor location, Borrmann type, tumor differentiation, TNM stage, N status and postoperative complications were independent predictors of OS (**Table 3**). In the PSM cohort, young age (15-39 years) was the independent risk factors for OS of GC patients after gastrectomy (HR 1.586, 95% CI 1.134-2.219; *p* = 0.007) (**Table 4**). Other independent predictors of OS included intraoperative blood transfusion, tumor location, Borrmann type and N status.

**Table 3:** Univariate and multivariate Cox regression analyses of overall survival after gastrectomy for gastric cancer before propensity score matching.

Variables		Univariate		Multivariate	
		HR (95% CI)	*p* value	HR (95% CI)	*p* value
Age	AYA vs. OA	1.087 (0.867-1.363)	0.467	NA	0.092
Sex	Female vs. Male	1.024 (0.881-1.191)	0.754		
Family history of cancer	Yes vs. No	0.657 (0.505-0.855)	0.002**	0.693 (0.532-0.904)	0.007**
Comorbid conditions	≥ 2 vs. < 2	1.259 (0.807-1.965)	0.31		
CEA	> 5 vs. ≤ 5μg/L	1.613 (1.352-1.925)	< 0.001***	1.23 (1.022-1.48)	0.029*
Operation time	> 300 vs. ≤ 300min	1.325 (1.139-1.541)	< 0.001***	NA	0.193
Gastrectomy type	Total vs. subtotal	1.945 (1.684-2.247)	< 0.001***	NA	0.294
Intraoperative blood transfusion	> 200 vs. ≤ 200ml	1.647 (1.418-1.912)	< 0.001***	1.426 (1.223-1.662)	< 0.001***
Ascites	Yes vs. No	1.448 (1.108-1.893)	0.007**	NA	0.158
Tumor size	> 5 vs. ≤ 5cm	2.076 (1.791-2.405)	< 0.001***	NA	0.136
Tumor site					
	Upper	Reference	< 0.001***	Reference	< 0.001***
	Middle	0.654 (0.525-0.815)	< 0.001***	0.673 (0.536-0.844)	0.001**
	Lower	0.604 (0.514-0.709)	< 0.001***	0.659 (0.557-0.78)	< 0.001***
	Whole	2.497 (1.803-3.458)	< 0.001***	1.115 (0.779-1.595)	0.552
Borrmann type					
	I	Reference	< 0.001***	Reference	< 0.001***
	II	0.537 (0.366-0.785)	0.001**	0.702 (0.477-1.033)	0.073
	III	1.112 (0.781-1.583)	0.556	0.857 (0.598-1.227)	0.399
	IV	2.704 (1.818-4.022)	< 0.001***	1.607 (1.059-2.438)	0.026**
Differentiation					
	Well	Reference	< 0.001***	Reference	< 0.001***
	Moderate	1.805 (1.025-3.18)	0.041**	1.042 (0.582-1.866)	0.89
	Poor	3.41 (1.967-5.913)	< 0.001***	1.706 (0.966-3.013)	0.066
TNM stage					
	I	Reference	< 0.001***	Reference	< 0.001***
	II	2.822 (2.155-3.694)	< 0.001***	1.782 (1.316-2.413)	< 0.001***
	III	6.797 (5.233-8.829)	< 0.001***	2.057 (1.406-3.009)	< 0.001***
T status					
	1	Reference	< 0.001***	Reference	0.156
	2	1.8 (1.227-2.638)	0.003**	NA	0.673
	3	4.008 (2.937-5.47)	< 0.001***	NA	0.043*
	4	5.316 (3.875-7.294)	< 0.001***	NA	0.144
N status					
	N0	Reference	< 0.001***	Reference	< 0.001***
	N1	2.225 (1.81-2.735)	< 0.001***	1.567 (1.243-1.977)	< 0.001***
	N2	3.963 (3.194-4.916)	< 0.001***	2.326 (1.69-3.201)	< 0.001***
	N3	6.693 (5.395-8.303)	< 0.001***	3.328 (2.382-4.651)	< 0.001***
Vascular invasion	Yes vs. No	1.911 (1.332-2.741)	< 0.001***	NA	0.761
Lymphatic invasion	Yes vs. No	1.435 (1.031-1.998)	0.032*	NA	0.376
Perineural invasion	Yes vs. No	1.625 (0.957-2.759)	0.072	NA	0.755
Lymphadenectomy type	< D2 vs. ≥ D2	1.091 (0.601-1.979)	0.775	NA	
Postoperative complications	Yes vs. No	1.47 (1.179-1.832)	0.001**	1.328 (1.062-1.661)	0.013*
Adjuvant chemotherapy	No vs. Yes	0.944 (0.815-1.092)	0.437		

AYA, adolescent and young adult; OA, older adult; HR, hazards ratio; CI, confidence interval; PSM, propensity score match; NA, not applicable; * p < 0.05; ** p < 0.01; *** p < 0.001.

**Table 4:** Univariate and multivariate Cox regression analyses of overall survival after gastrectomy for gastric cancer after propensity score matching.

Variables		Univariate		Multivariate	
		HR (95% CI)	*p* value	HR (95% CI)	*p* value
Age, year	AYA vs. OA	1.41 (1.021-1.948)	0.037*	1.586 (1.134-2.219)	0.007**
Sex	Female vs. Male	1.101 (0.797-1.519)	0.56		
Family history of cancer	Yes vs. No	0.664 (0.382-1.153)	0.145		
Comorbid conditions	≥ 2 vs. < 2	NA	NA		
CEA	> 5 vs. ≤ 5μg/L	1.469 (0.856-2.523)	0.163		
Operation time	> 300 vs. ≤ 300min	1.153 (0.803-1.655)	0.441		
Gastrectomy type	Total vs. subtotal	2.349 (1.692-3.261)	< 0.001***	NA	0.115
Intraoperative blood transfusion	> 200 vs. ≤ 200ml	1.561 (1.119-2.178)	0.009**	1.963 (1.386-2.782)	< 0.001***
Ascites	Yes vs. No	1.87 (1.036-3.375)	0.038*	NA	0.986
Tumor size	> 5 vs. ≤ 5cm	2.416 (1.744-3.347)	< 0.001***	NA	0.69
Tumor site					
	Upper	Reference	< 0.001***	Reference	0.005**
	Middle	0.864 (0.526-1.421)	0.566	0.951 (0.569-1.588)	0.847
	Lower	0.688 (0.444-1.067)	0.095	0.727 (0.466-1.135)	0.161
	Whole	4.067 (2.16-7.657)	< 0.001***	2.269 (1.147-4.489)	0.019*
Borrmann type					
	I	Reference	< 0.001***	Reference	< 0.001***
	II	1.2 (0.421-3.418)	0.733	1.121 (0.39-3.217)	0.832
	III	2.168 (0.79-5.948)	0.133	1.394 (0.497-3.91)	0.528
	IV	6.792 (2.35-19.632)	< 0.001***	3.663 (1.226-10.946)	0.02*
Differentiation					
	Well	Reference	0.129		
	Moderate	3.533 (0.468-26.663)	0.221		
	Poor	4.969 (0.694-35.562)	0.11		
TNM stage					
	I	Reference	< 0.001***	Reference	0.938
	II	1.647 (0.979-2.773)	0.06	NA	0.975
	III	4.558 (2.77-7.5)	< 0.001***	NA	0.824
T status					
	1	Reference	< 0.001	Reference	0.759
	2	1.411 (0.691-2.882)	0.344	NA	0.289
	3	2.524 (1.389-4.584)	0.002**	NA	0.541
	4	3.715 (2.018-6.84)	< 0.001***	NA	0.889
N status					
	N0	Reference	< 0.001	Reference	< 0.001***
	N1	1.757 (1.11-2.781)	0.016*	2.085 (1.291-3.367)	0.003**
	N2	4.003 (2.501-6.407)	< 0.001***	4.261 (2.607-6.963)	< 0.001***
	N3	6.193 (3.869-9.912)	< 0.001***	6.765 (4.081-11.214)	< 0.001***
Vascular invasion	Yes vs. No	2.793 (1.366-5.713)	0.005**	NA	0.353
Lymphatic invasion	Yes vs. No	1.154 (0.366-3.641)	0.808		
Perineural invasion	Yes vs. No	1.919 (0.709-5.192)	0.2		
Lymphadenectomy type	< D2 vs. ≥ D2	0.719 (0.178-2.908)	0.643		
Postoperative complications	Yes vs. No	1.178 (0.55-2.523)	0.673		
Adjuvant chemotherapy	No vs. Yes	1.026 (0.740-1.423)	0.877		

AYA, adolescent and young adult; OA, older adult; HR, hazards ratio; CI, confidence interval; PSM, propensity score match; NA, not applicable; * p < 0.05; ** p < 0.01; *** p < 0.001.

### 3.4 Prognostic analyses of OS among AYA and OA patients

**Figure 4:**
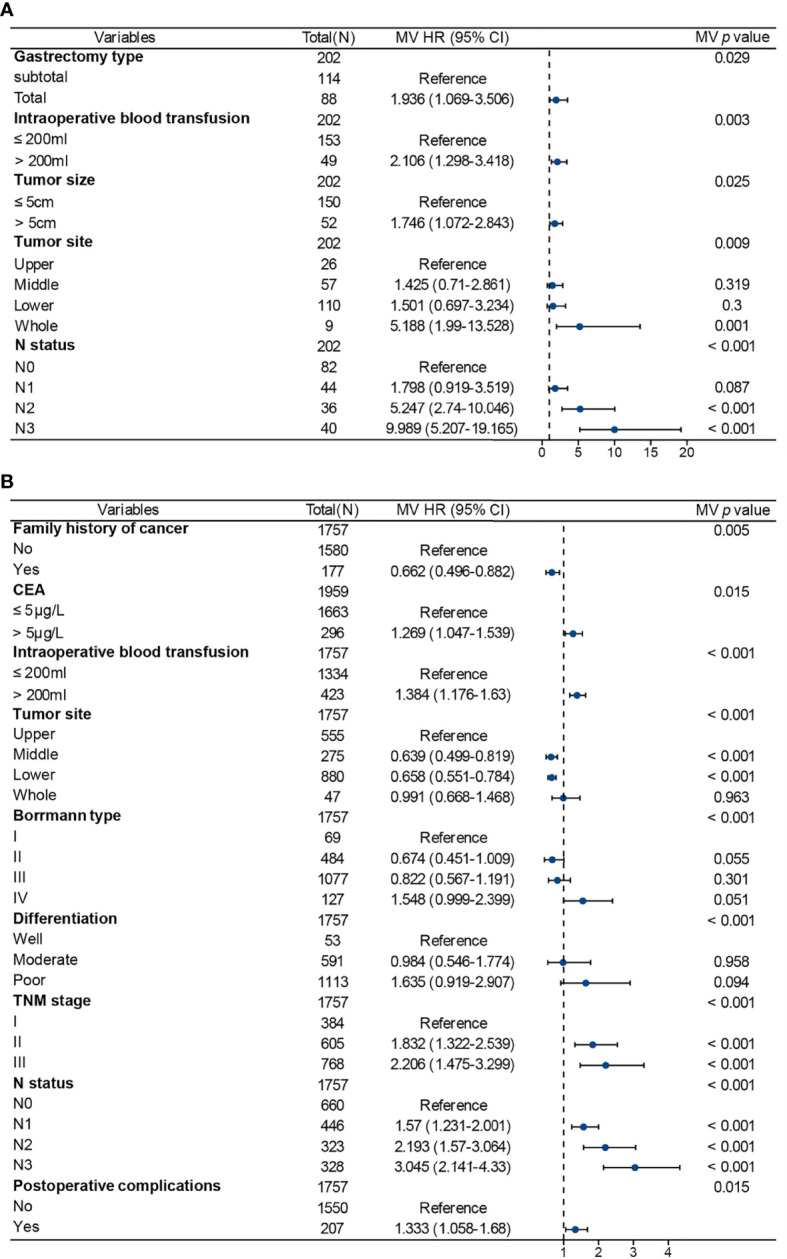
Multivariate Cox regression analysis for factors associated with prognosis in patients after gastrectomy for gastric cancer. **(A)** Adolescents and young adults. **(B)** Older adults. MV, multivariable; HR, hazards ratio; CI, confidence interval.

In the sub-analysis of the AYA patient cohort, gastrectomy type, intraoperative blood transfusion, tumor size, tumor location and N status were independent predictors of OS (**Figure 4A**). In the OA patients, family history of cancer, CEA level, intraoperative blood transfusion, Borrmann type, tumor location, tumor differentiation, TNM stage, N status and postoperative complications were independent predictors of OS (**Figure 4B**). There were some common independent predictors of OS between AYA patients and OA patients, including intraoperative blood transfusion, tumor location and N status.

## 4 Discussion

Cancer was the fourth leading cause of death in AYAs and contributed substantially to the overall disease burden of AYAs globally (21). Despite rapid advances in the understanding of cancer in AYAs in recent years, there are still few studies on the clinicopathological features and prognosis of AYA patients with GC. We conducted a retrospective cohort study to analyze the clinicopathological characteristics, as well as perioperative outcomes and long-term outcomes, following gastrectomy for GC in 202 AYA patients and 1757 OA patients.

Consistent with previous studies (5–9, 22), our study found that the proportion of females was significantly higher in AYA patients than in OA patients. In terms of clinical manifestations, our study indicated that AYA patients with GC are less likely to present manifestations such as abdominal distension, dysphagia, and weight loss compared to OA patients. This suggests that the onset of GC in AYA patients may be more covert, which could explain the higher proportion of AYA patients with advanced-stage cancer reported in other studies (2, 23, 24).

Our study found that AYA patients had a higher proportion of normal CEA levels than OA patients, which is consistent with previous studies (25). It has been reported that the higher positive rate of serum CEA in GC patients is related to the tumors located in the upper third of the stomach (26). In our study, the proportion of tumors located in the upper third of the stomach was lower in AYA patients than in OA patients, in agreement with previous studies (22, 24). Therefore, we speculate that the lower serum CEA positivity rate in AYA patients compared to OA patients may be attributed to the lower proportion of AYA patients with tumors located in the upper third of the stomach. It is well established that long-term gastroesophageal reflux disease (GERD) can lead to chronic inflammation and mucosal damage in the stomach, which may result in intestinal metaplasia and dysplasia, the precursor to GC (27). The incidence of GERD tends to increase with age (28, 29), which may explain the higher proportion of tumors located in the upper third of the stomach among OA patients compared to AYA patients.

Previous studies have reported a higher proportion of diffuse-type tumors in young patients compared to middle-aged patients (2). In our study, there was no significant difference in tumor type between AYA and OA patients. However, we found a significantly higher proportion of poorly differentiated tumors in AYA patients compared to OA patients, which is in line with previous studies (2, 8, 22, 24). Additionally, perineural invasion was observed more frequently in AYA patients as well. There is evidence suggesting that, for most types of cancer, AYAs are more likely to develop metastasis compared to middle-aged and elderly patients (30). This indicates that the biological behavior of GC in AYA patients may be more aggressive.

When examining perioperative outcomes, it was observed that AYA patients had a lower incidence of postoperative complications compared to OA patients, which is consistent with previous study (2). In addition, the length of postoperative hospital stay of AYA patients was also shorter than that of OA patients, which may be attributed to the stronger recovery ability of AYA patients. However, we found no statistically significant differences in postoperative 30-day and 90-day mortality between the two groups, either before or after PSM. In terms of long-term outcomes, AYA patients demonstrated comparable OS to OA patients before PSM, in agreement with previous studies (2, 8, 22, 31). However, studies have suggested that comorbid conditions and postoperative complications are associated with poorer prognosis (32–34), and both the proportions of comorbid conditions and postoperative complications were lower in AYA patients compared to OA patients in our study. The lower incidence of comorbid conditions and postoperative complications in AYA patients may be the reason for no significant difference in OS between the two groups in the entire cohort. In addition, in our study, the proportion of AYA patients receiving adjuvant chemotherapy after surgery was higher than that of OA patients. Previous studies have demonstrated that adjuvant chemotherapy can improve the prognosis of GC patients (35, 36). Thus, this difference in the proportion of patients receiving adjuvant chemotherapy may be another reason why there was no significant difference in OS between AYA patients and OA patients before PSM.

After balancing the factors which may affect the prognosis between the two groups by PSM, the OS of AYA patients was observed to be inferior to that of OA patients. Furthermore, the results of multivariate Cox regression analysis also suggested that young age (15-39 years) was an independent risk factor for OS in patients with GC after gastrectomy.

In addition, we further analyzed the variables associated with OS in AYA and OA patients, respectively. The results showed that there were significant differences between the two groups in independent risk factors of OS. This may be due to the difference in tumor biology between AYA patients and OA patients. Clinicopathological variables associated with poorer OS in AYA patients with GC included total gastrectomy, intraoperative blood transfusion > 200mL, tumor size > 5cm, tumor diffuse in the whole stomach, and advanced N status. Many previous studies have also reported that these variables are associated with the prognosis of patients with GC after gastrectomy (37–39). Liu et al. also reported that tumor location and N status were independent predictors for the prognosis in young patients with GC (22). Therefore, for AYA patients with the above clinicopathological features, it may be necessary to strengthen postoperative surveillance to improve the prognosis.

AYAs have unique epidemiology, clinical care needs, and societal implications compared with children and adults (21). Additionally, cancers in AYAs also tend to be biologically distinct from patients in other age groups and may benefit from different treatments (40). Thus, there is a need for further research to investigate the unique biological and clinicopathological characteristics, as well as prognostic factors, specific to AYAs with GC. Such investigations would facilitate the development of targeted interventions for improving the prognosis of AYA patients with GC.

This study has several limitations. Firstly, it is a retrospective study conducted at a single center, which may have inherent potential bias, and carefully designed randomized clinical trials should be conducted to avoid statistical bias. Secondly, although factors between the two groups were balanced by PSM, the relatively small number of AYA patients (N = 202) may have limited statistical power. However, it is known that this study is currently the largest comparative analysis of clinicopathological characteristics and postoperative prognosis of AYA and OA patients in China. Additionally, all patients in this study received treatment in China. Therefore, it is crucial to externally validate the data obtained from this study in Western patients to ensure that the findings of the study can be generalized to a broader patient population.

## 5 Conclusions

Compared to OA patients, AYA patients with GC tend to have fewer clinical manifestations, a higher prevalence of females, poorer differentiation, normal CEA levels, a lower proportion of tumors located in the upper third of the stomach, a greater likelihood of perineural invasion, and a higher rate of receiving adjuvant chemotherapy. The perioperative outcomes of AYA patients with respect to postoperative complications and length of postoperative hospital stay were found to be superior to those of OA patients, but the OS was inferior to that of OA patients. Gastrectomy type, intraoperative blood transfusion, tumor size, tumor location, and N status were identified as independent predictor of prognosis in AYA patients.

## Data availability statement

The raw data supporting the conclusions of this article will be made available by the authors, without undue reservation.

## Ethics statement

Written informed consent was obtained from the individual(s) for the publication of any potentially identifiable images or data included in this article.

## Author contributions

Formal analysis and writing original draft: H-WC. Data curation and writing original draft: X-YC. Software and writing original draft: XL. Methodology: C-CD. Visualization: BB. Project administration: Y-LH. Writing, review, and editing: M-YH. Supervision, project administration, and funding acquisition: C-HZ. All authors contributed to the article and agreed to the published version of the manuscript.
